# A Comparative Evaluation of the Effects of N95 and Surgical Facemasks on Pregnant Women Performing Moderate-Intensity Exercise: A Prospective Randomized Study

**DOI:** 10.7759/cureus.45776

**Published:** 2023-09-22

**Authors:** Ubong B Akpan, Chinyere J Akpanika, Udeme Asibong, Mabel Ekott, Saturday Etuk

**Affiliations:** 1 Department of Obstetrics and Gynaecology, University of Calabar Teaching Hospital, Calabar, NGA; 2 Department of Family Medicine, University of Calabar Teaching Hospital, Calabar, NGA

**Keywords:** exercising pregnants wearing masks, surgical facemask, n95 mask, tachypnea, tachycardia, oxygen saturation

## Abstract

Background and objective

A facemask is often indicated for the control of the spread of airborne pathogens. At the peak of the COVID-19 pandemic, there was mass enforcement of mask use across the globe. Pregnant women were not excluded. While several studies have been conducted to evaluate and compare the efficacy of various mask types, data on their effects on pregnant women during exercise are scarce. The objective of this study was to evaluate and compare the effects of N95 and surgical facemasks on the cardiopulmonary functions of pregnant women during moderate-intensity exercise.

Methods

A prospective randomized study was conducted among 104 healthy women with advanced singleton pregnancies performing moderate-intensity exercise wearing either surgical or N95 masks during routine antenatal care. Their respiratory rates were counted, and arterial oxygen saturation (SPO_2_) and radial pulses (heart rates) were recorded with a mobile digital pulse oximeter at baseline and after 30 minutes of exercise. The mean values were calculated. Data analysis was done using Statistical Product and Service Solutions (SPSS, version 25; IBM SPSS Statistics for Windows, Armonk, NY). An independent t-test was used to compare the mean SPO_2_ and radial pulse between the two groups. Chi-square was used to examine differences in categorical variables. The level of significance was set at 0.05.

Results

Their demographic profiles and measured baseline parameters were comparable. Following a 30-minute exercise, the N95 mask group had lower mean SPO_2_ compared to the surgical mask group (95.5% versus 97.0%; P=0.028, 95%CI; -2.607 to 0.15). Further, the N95 group recorded a higher mean heart rate than the surgical mask group ((97.23 b/m versus 95.02b/m, respectively, mean difference (MD)=2.212, P=0.021, 95%Cl: 1.249-3.672). The mean respiratory rates were also higher among women in the N95 mask group (32.1 c/m versus 29.08 c/m, MD=3.018, 95%CI: 1.392-4.662, P=0.001).

Conclusion

The study, comparing the relative effects of the surgical and N95 facemask on the cardiorespiratory functions of exercising pregnant women, findings suggest that surgical facemasks may be better tolerated in advanced pregnancy when performing routine antenatal aerobic exercise in comparison with N95 masks.

## Introduction

There has been genuine concern about the potential mask-induced maternal hypoxia and the risks to the fetus. This is related to the potential effect of a protective face piece in obstructing gaseous exchange [1,2]. The physiological changes in pregnancy increase the haemodynamic burden on cardio-pulmonary systems [3]. These changes are adaptations to meet the metabolic demands of the mother and the developing fetus [4-6]. Some respiratory changes may lead to a reduction in lung volumes due to splinting of the diaphragm by the enlarged gravid uterus, which may affect the woman’s ability to tolerate stressful events [6]. A previous study on pregnant women has shown that wearing a facemask at rest did not significantly reduce arterial oxygen saturation (SPO_2_) and heart rates when compared with non-pregnant controls [7]. Another study also found that wearing surgical facemasks during a nonstress test for fetal surveillance did not significantly affect fetal parameters, such as heart rate reactivity, variability, and decelerations [8]. However, data on the effects of surgical and N95 masks on ambulating pregnant women are either lacking or inconclusive and deserve further randomized studies.

Studies have shown that pregnant women who exercise regularly during pregnancy have reduced incidences of gestational diabetes mellitus, lower rates of caesarean delivery, early postpartum recovery, and lower incidence of preeclampsia [9,10]. Physical activity during pregnancy is also considered an essential factor for the prevention of postpartum depression [11]. American College of Sports Medicine recommends that women with uncomplicated pregnancies should be encouraged to engage in aerobic and strength-conditioning exercises during pregnancy [12]. Low-to-moderate exercise for 30 minutes a day is recommended [12]. Furthermore, a study examining the effects of exercise among obese pregnant women demonstrated a moderate reduction in weight gain with no adverse outcomes [13]. The WHO and CDC recognized the added benefits of exercising at a greater intensity to improve cardiorespiratory fitness and to reduce the risk of mortality and morbidity [11]. Physical activity does not increase a woman’s risk of miscarriage, low birth weight, or preterm delivery [11].

Furthermore, preliminary data suggest that exercise during pregnancy has a lifelong protective effect, resulting in a reduced cardiovascular risk profile in perimenopause women [13]. Maternal physical exercise is also beneficial for the fetus, resulting in a lower risk of fetal macrosomia and consequently improved cardiovascular health of the infant [14].

In our tertiary health facility, routine antenatal programs include 30 minutes of moderate-intensity aerobic exercise, except when there are contraindications. This is usually done in groups during scheduled antenatal care (ANC) visits and is supervised by trained health personnel. However, this aspect of the ANC program was suspended during the peak of COVID-19 due to uncertainty concerning possible maternal and fetal complications when exercising with a facemask. Similarly, many ‘contact sports’ were prohibited in many societies for fear of infection spread. This prompted interest in this study.

Facemasks were previously used by healthcare workers or factory workers who were exposed to the risk of inhalation of dust, dangerous fumes, or pathogens, as a barrier to break the transmission of these hazardous materials. At the peak of the COVID-19 pandemic, the use of face masks proved to be a major preventive strategy, necessitating mass enforcement across the globe. Masks were also required to be worn while performing daily occupational tasks, which may involve moderate exertion equivalent to low- or moderate-intensity exercise. In many settings, 50% or more of the proportion of the workforce are females, and about 10% of these women may be pregnant [15].

A previous study on ambulating pregnant healthcare workers showed a possible negative impact of N95 masks on the participants with regard to changes in their respiratory functions [16]. However, this study enrolled non-pregnant women as controls and did not compare the safety profile of an alternative face piece. Findings from recent studies suggest that exercising with mask-on do not significantly cause respiratory or cardiac complication in healthy young non-pregnant individuals [17,18]. While studies on the impact of surgical, cloth, and N95 masks on exercising pregnant women have yielded contradicting results [19,20], there is a need for more prospective randomized comparative trials for more high-quality evidence and recommendations with regard to safety in pregnancy.

In view of all these, it is necessary to establish the safety or otherwise of N95 masks in comparison with surgical facemasks on the respiratory and cardiac activity in exercising pregnant women. Findings from this study may help in formulating guidelines on mask use among ambulating pregnant women in the face of emerging air-borne pathogenic infections.

## Materials and methods

Research setting

The study was conducted at the University of Calabar Teaching Hospital (UCTH) from the 1st of June 2021 to the 31st of September 2021. 

Research design and research population

This was a prospective randomized comparable study where the patients were randomized into two groups using computer-generated random numbers. A total of 104 pregnant women attending an antenatal clinic (ANC) were randomized into two groups, 52 women in each arm. Group 1 was given N95 face masks, while Group 2 was given surgical face masks. Participants were instructed to wear a nose mask for at least 10 minutes in a resting position. The mask was examined for proper application to ensure that the mouths and nostrils were covered. The baseline respiratory rates were noted, while the SPO_2_ and maternal radial pulses were measured with a mobile digital pulse oximeter. The mobile pulse oximeter was firmly secured to their left thumb. The women were then instructed to perform the same pattern of moderate-intensity aerobic exercise (rhythmic dancing and body flexion and extension) for 30 minutes as routinely done during regular ANC visits. The values of pulse and oxygen saturation recorded were done continuously with the pulse oximeter, while the measurement of respiratory rates was done within two minutes of the end of the exercise. The exercise instructors were trained health workers who were educated on the purpose of the study.

Standard surgical masks and N95 masks from the same manufacturers were used. The finger probe of the pulse oximeter was applied to the left thumb and recorded electronically and continuously during the time of exercise. The recording and measurements of respiratory rates were done by trained nurses and midwives. The mean of the recorded parameters for each participant was calculated. Every participant performed the same exercise for the same duration of time. The study was conducted in a well-ventilated area of the clinic. Women who felt significant discomfort were advised to discontinue at any time. The women were also asked to mention any side effects they noticed.

A structured questionnaire was used to obtain information on the socio-demographic, obstetric, and medical profiles of the participants. Their body mass index (BMI) was calculated from weight and height and recorded in kg/m^2^.

The pulse oximeters used in this study were manufactured by ACARE Technology Company Limited with model number AH-M1. A pulse oximeter can detect the oxygen saturation of haemoglobin quickly and in an accurate and reliable way. Pulse oximetry combines oximetry and plethysmography to measure SPO_2_ non-invasively [21]. According to Lambert Beer’s law, oxygenated and reduced hemoglobin absorb red and infrared light differently. Oxyhaemoglobin absorbs more infrared light, whereas reduced haemoglobin absorbs more red light. The rate of absorption at the red and infrared wavelengths is analyzed by oximetry to give the oxygen saturation of arterial pulsations [21].

The normal oxygen saturation in a healthy individual is between 95% and 100% [22]. Their pulse rates were also classified as normal (<100 beats per minute) and tachycardia (100 b/m and above) [23].

**Figure 1:**
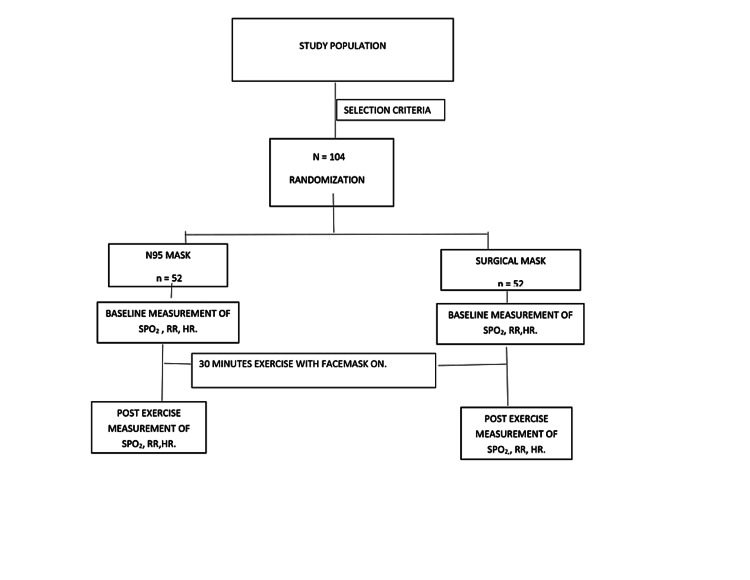
Flowchart summarizing the research protocol. SPO2: oxygen saturation, RR: respiratory rate, HR: heart rate

The participants were screened for medical or obstetric complications. The fetal heart rates were examined before and after the test. Oxygen cylinders with connections were made available in case of prolonged hypoxemia. The maternity emergency unit was notified in case of an obstetric emergency. The women were instructed to discontinue at any time if they experienced any problem during the 30-minute exercise period. Figure 1 is a flowchart summarizing the research protocol.

Classification of exercise intensity

In this study, the level of exercise intensity was classified based on the CDC activity classification tables. Moderate-intensity exercise is considered to be associated with an energy expenditure of 3.5-7 kcal per minute and performed within the duration of 20-50 minutes, equivalent to three to six exercise metabolic rates (METs) [24]. Activities in this group include aerobic dancing, swimming, playing table tennis, and badminton. It also includes occupational events such as tree planting, weeding, stacking wood, and raking for that period of time [24]. The ratio of exercise metabolic rate, MET, is defined as the energy expenditure for sitting quietly, which, for the average adult, approximates 3.5 mL of oxygen uptake per kilogram of body weight per minute (1 MET=1.2 Kcal/min for 70-kg individuals). It is recommended that a healthy individual undertakes 150 minutes of moderate- to vigorous-intensity physical activity each week [25].

Inclusion criteria

Healthy pregnant women, ages 18-40 years, with singleton pregnancy, who presented for routine ANC between the gestational ages of 30 and 36 weeks.

Exclusion criteria

The following group of women was excluded from the study: history of vaginal bleeding in the current pregnancy, respiratory or cardiovascular problems, history of preterm labour or intrauterine growth restriction, anaemia in pregnancy (packed cell volume {PCV} <30%), abnormal vital signs values, unexplained chest pain, calf pain or swelling, liquor drainage, and abdominal pain.

Ethical issues

Approval was obtained from the UCTH Health Research Ethics Committee (protocol assigned number UCTH/HREC/33/563). Participation was voluntary. A written informed consent was obtained from every woman before enrollment. Confidentiality was maintained. Emergency care was made available where needed by any participant during the course of the exercise period who experienced any form of complication.

Data analysis

Data analysis was done using Statistical Product and Service Solutions (SPSS, version 25; IBM SPSS Statistics for Windows, Armonk, NY). The values of scale and continuous data were presented as mean ± standard deviation. An independent t-test was used to compare the means of SPO_2_ and radial pulse between the two groups. Chi-square was used to examine differences in categorical variables. The level of significance was set at p≤0.05.

## Results

A total of 104 participants, 52 in each group, were included in the analysis. The mean ages of the women in the two groups were similar: 30.18 ± 4.990 and 29.06 ± 4.400 (p=0.184) for the N95 and surgical face mask groups, respectively. Similarly, their mean gestational age in weeks and mean parity, as well as their mean baseline SPO_2_ and pulse rates, were comparable, as shown in Table 1.

**Table 1:** Maternal demographic and baseline characteristics. SD: Standard Deviation

Variables	Mask type	Mean	±SD	P value
Body Mass Index	N95 (n=52)	30.18	±4.990	0.184
	SURGICAL (n-52)	29.06	±4.400	
Age (years)	N95(n-52)	30.52	±4.509	0.166
	SURGICAL(n-52)	31.94	±5.812	
Gestational age(weeks)	N95(n=52)	32.12	±2.628	0.331
	SURGICAL(n=52)	31.58	±2.986	
Parity	N95 (n=52)	2.0	±1.029	0.418
	SURGICAL(n=52)	1.85	±0.874	
Baseline SPO_2_	N95 (n=52)	98.71	±2.080	0.152
	SURGICAL (=52)	99.15	±0.751	
Baseline respiratory rate	N95 (n-52)	24.13	±2.417	0.578
	SURGICAL (n=52)	23.85	±2.838	
Baseline Pulse rates	N95(n=52)	87.98	±9.001	0.881
	SURGICAL(n=52)	87.73	±7.988	

The study showed that the women with advanced singleton pregnancy, performing routine aerobic antenatal exercise, can tolerate surgical or N95 masks on exercise as none of the participants withdrew during the 30-minute study. The overall mean SPO_2_ and the mean heart rate of all the participants were within the theoretical normal range (optimal normal range: 95%-100% for SPO_2_ and 60-100 for heart rate). The mean SPO_2_ in the N95 mask group was 95.52±3.976 compared to 97.01±2.067 for the surgical mask group (p=0.028). The mean heart rate was also significantly higher in the N95 mask group (97.23± 8.252 b/m versus 95.02±9.494 b/m; p=0.021). Although there was no significant difference in the number of women who experienced unwanted side effects such as headache, dizziness, and breathlessness, the women in the N95 group had higher values of respiratory rates compared to the surgical mask group. The postexercise findings are summarized in Table 2.

**Table 2:** Postexercise cardio-respiratory values. *statistically significant (p value<0.05) MD: mean difference, OR: odd ratio, CI: confidence interval, c/m: cycles per minute

Past Exercise Values	Mask Type	Mean ±SD	P value	MD/OR	95% CI	
					Lower	Upper
Mean oxygen saturation (SPO_2_)(%)	N95 (n=52)	95.52 ± 3.976	0.028*	1.397	-2.612	0.152
	Surgical (n=52)	97.1 ± 2.067				
Mean heart rate (HR)	N95 (n=52)	97.23 ± 8.252	0.021*	2.212	1.249	5.672
	Surgical (n=52)	95.02 ± 9.494				
Respiratory rate (RR) (c/m)	N95 (n=52)	32.10	0.001*	3.019	1.392	4.662
	Surgical (n=52)	29.08				
Tachycardia	N95 (n=52)	19 (36.53%)	0.339	1.135	0.767	1.679
	Surgical (n=52)	16 (30.77%)				
Hypoxic symptoms	N95 (n=52)	7 (13.5%)	0.380	1.193	0.708	2.010
	Surgical (n=52)	5 (9.6%)				

On bivariate analysis, the study showed the relationship between maternal BMI and the changes in SPO_2_ and heart rate during exercise. There was a weak negative relationship between maternal BMI and SPO_2_ (r=-0.228). This implies that, for every 1 kg/m^2^ change in BMI, the SPO_2_ drops by 0.228% during moderate-intensity exercise. Additionally, the analysis showed that the maternal heart rate increases by 0.249 b/m for every 1 kg/m^2^ change in maternal BMI (R=O.249).

## Discussion

The study examines the relative effects of two of the most utilized protective facemasks on the cardio-respiratory parameters in pregnancy during exercise. The results show that women in both groups exhibited a fair tolerance to moderate-intensity exercise with either surgical or N95 masks. This is evidenced by no record of any unwanted event that necessitated withdrawal from the task during the 30 minutes of aerobic exercise among the study participants.

In keeping with previous findings [17,20], this study shows marked changes in the maternal pulse rate, respiratory rate, and oxygen saturation between preexercise and postexercise values. The differences in mean SPO_2_, heart rate, and respiratory rate in pre- and postexercise values were statistically significant (p<0.05). These changes during exercise are considered beneficial in healthy individuals as they promote physical and mental fitness and reduce incidences of these major organ disorders, leading to overall improvement in the lifespan of people exercising regularly. Thus, the CDC and WHO recommend low- to moderate-intensity exercise of 30 minutes per day during pregnancy [11,12].

In the present study, the women in the surgical mask group had higher values of SPO_2_ during exercise compared to women in the N95 mask groups, and the difference was statistically significant (p=0.028). This finding is in keeping with previous reports [17,19,26]. Breathing through N95 mask materials has been shown to impede gaseous exchange. Roberge et al. [27] reported that breathing through the N95 mask during low-intensity work reduced expired oxygen concentration by 32% with no significant change in breathing frequency among pregnant healthcare workers. In a systematic review of N95 respirator masks during pregnancy, Roeckner et al. [2] found that breathing through the mask also imposes an additional workload on the metabolic system of pregnant women. They concluded that the benefits of using N95 masks to prevent infectious diseases should be weighed against the potential respiratory consequences associated with extended use.

Majeck et al. [17] compared preexercise and postexercise values of respiration and heart rate. They found that a short-term exercise performed by these young people using protective masks did not affect oxygen saturation, heart rate, blood pressure, and respiratory rates. These were contrary to our findings. The present study was performed among women with advanced pregnancy (third trimester). Another contradicting report by Toprak et al. [26] found that the mask tolerance of people using respiratory mass was significantly higher than those using surgical masks in pregnancy. In this study, the parameters were measured before and after a nonstress test, and there was no significant energy-consuming event such as aerobic exercise. This may not be extrapolated to the routine workload undertaken by women in pregnancy.

The mean pulse rates in both groups were within normal limits, indicating that the cardiovascular status of the majority of the subjects was not negatively impacted by wearing either type of facemasks during low- to moderate-intensity work. However, women in the N95 mask group recorded higher values of heart rates, and the proportion of subjects in that group whose heart rate exceeded 100 b/m (tachycardia) during exercise was significantly higher than those in the surgical mask group. This is in keeping with a previous report [27]. Reduced arterial oxygen saturation during exercise leads to an increase in cardiac output to enhance tissue perfusion [23]. This increase in cardiac output is due partly to an increase in heart rate.

The study showed a slight (non-significant) negative correlation (r=-0.228) between oxygen saturation and maternal BMI and a positive correlation between BMI and heart rates (r=0.249). This implies that, for every 1 kg/m^2^ change in maternal BMI, oxygen saturation is likely to drop by 0.228, while the heart rate is likely to increase by 0.249 b/m during moderate exercise. This compensatory adjustment in oxygen saturation and heart rates with respect to the BMI during exercise reflects the increased need to maintain cellular aerobic respiration by increasing oxygen uptake to mitigate the impact of low SPO_2_ on maternal and fetal health [27-29]. While our study did not include women with severe obesity (BMI of 40 and above), there is a chance that marked maternal obesity may worsen maternal cardio-respiratory parameters during exercise with a facemask in pregnancy [23].

The subjects were asked about any unwanted feelings exercising with the masks on. Only 13.5% in the N95 and 9.6% in the surgical mask (p=0.380) reported mild symptoms of headache, dizziness, and breathlessness. These symptoms resolved without treatment. In contrast to this finding, previous studies on healthcare workers using N95 masks found that a significant proportion of them developed persistent headaches, dizziness, confusion, and visual problems with the use of the mask, and those who had pre-existing headaches reported worsening conditions that required pain medication [27,28]. Using a double surgical mask may not offer added protection but may worsen the hypoxic symptoms [28].

Strengths and limitations of the study

The major strength of this study was that the two types of masks studied are universally commonly available standard protective face masks. In most health facilities, these are the types of masks that people are allowed to wear in terms of hygiene purposes. Therefore, the findings from this study reflect the true situation in the general population. One of the main limitations is that this study was conducted among apparently healthy pregnant women. As such, the findings may not be generalized in people with morbid obesity or chronic cardiovascular and lung diseases. Additionally, the small sample size suggests the need for a larger multicenter study, which is being planned. A control group of pregnant women who did not use facemasks should have been used to compare with the ones who used facemasks, and this is intended to be done in further research studies.

## Conclusions

The findings in this study suggest that women with advanced singleton gestation may perform low-to-moderate intensity exercise with either N95 or surgical masks for 30 minutes without significant harmful effects. It is, therefore, recommended that pregnant women be encouraged to use a facemask when exercising during the pandemic or any airborne disease outbreak. In pregnancy, wearing a surgical facemask during exercise is associated with milder cardio-respiratory changes compared to N95 masks. additionally, working-class women involved in strenuous occupations may use a mask in the settings of concern of infection spread.
